# Radiotherapy alters expression of molecular targets in prostate cancer in a fractionation- and time-dependent manner

**DOI:** 10.1038/s41598-022-07394-y

**Published:** 2022-03-03

**Authors:** Iris Eke, Molykutty J. Aryankalayil, Michelle A. Bylicky, Adeola Y. Makinde, Lance Liotta, Valerie Calvert, Emanuel F. Petricoin, Edward E. Graves, C. Norman Coleman

**Affiliations:** 1 Department of Radiation Oncology, Center for Clinical Sciences Research (CCSR), Stanford University School of Medicine, 269 Campus Dr., Room 1260, Stanford, CA 94305 USA; 2 Radiation Oncology Branch, Center for Cancer Research, National Cancer Institute, National Institutes of Health, Bethesda, MD 20892 USA; 3 Center for Applied Proteomics and Molecular Medicine, George Mason University, Manassas, VA 20110 USA; 4 Radiation Research Program, National Cancer Institute, National Institutes of Health, Rockville, MD 20850 USA

**Keywords:** Radiotherapy, Targeted therapies, Cell death, Cell signalling, Mechanisms of disease, Post-translational modifications

## Abstract

The efficacy of molecular targeted therapy depends on expression and enzymatic activity of the target molecules. As radiotherapy modulates gene expression and protein phosphorylation dependent on dose and fractionation, we analyzed the long-term effects of irradiation on the post-radiation efficacy of molecular targeted drugs. We irradiated prostate cancer cells either with a single dose (SD) of 10 Gy x-ray or a multifractionated (MF) regimen with 10 fractions of 1 Gy. Whole genome arrays and reverse phase protein microarrays were used to determine gene expression and protein phosphorylation. Additionally, we evaluated radiation-induced pathway activation with the Ingenuity Pathway Analysis software. To measure cell survival and sensitivity to clinically used molecular targeted drugs, we performed colony formation assays. We found increased activation of several pathways regulating important cell functions such as cell migration and cell survival at 24 h after MF irradiation or at 2 months after SD irradiation. Further, cells which survived a SD of 10 Gy showed a long-term upregulation and increased activity of multiple molecular targets including AKT, IGF-1R, VEGFR2, or MET, while HDAC expression was decreased. In line with this, 10 Gy SD cells were more sensitive to target inhibition with Capivasertib or Ipatasertib (AKTi), BMS-754807 (IGF-1Ri), or Foretinib (VEGFR2/METi), but less sensitive to Panobinostat or Vorinostat (HDACi). In summary, understanding the molecular short- and long-term changes after irradiation can aid in optimizing the efficacy of multimodal radiation oncology in combination with post-irradiation molecularly-targeted drug treatment and improving the outcome of prostate cancer patients.

## Introduction

Although the 5-year survival rates for prostate cancer are more than 90% it is still one of the leading causes of cancer-related death among men in the United States due to its high incidence^1^. Approximately 1 in 8 men is diagnosed with prostate cancer at one point in his life and 1 in 41 men will die of it. Most patients have local disease but around 16% of prostate cancers have already spread to regional lymph nodes or other organs at the time point of diagnosis^2^. Between 20 and 30% of patients will have tumor relapse after curative treatment which can be either local or metastatic recurrence^3^. Interestingly, it has also been shown that local recurrence can occur by tumor cells originating from metastases repopulating the primary tumor site^4^. Multiple factors play a role in disease prognosis and guide therapy decisions such as patient age, Gleason score, and PSA level^5^. The evolving treatment options for primary prostate cancer are surgery and a variety of radiotherapeutic options which can be used alone or in combination. These regimens include standard fractionated, moderately hypofractionated^6^, or ultrahypofractionated (such as stereotactic ablative radiotherapy—SABR, also known as stereotactic body radiation therapy—SBRT) external beam radiotherapy in addition to temporary high dose or permanent low dose rate brachytherapy through implantation of radioactive sources into the malignant tissue. Proton therapy is also used but proton biology is not the topic of this report. Especially for patients with disease recurrence after radiation therapy, there is a high need for additional treatment options, since the possibilities to re-irradiate are often limited.

In recent years, targeted therapy was implemented into multimodal cancer treatment regimens generally used before or simultaneously with radiation^7^. The underlying hypothesis is that while conventional chemotherapy impacts proliferation and survival of both malignant and normal tissue, targeted therapeutics aim to exploit the abnormal molecular signaling often found in cancer cells and to target and kill tumors more specifically^7–9^.

Targeted therapy is often directed against kinases overexpressed in cancer driving survival and proliferation such as receptor tyrosine kinases (RTK) and associated molecules. Pre-clinical studies show that inhibition of the epidermal growth factor receptor (EGFR) reduces tumor growth and results in radiosensitization of different cancer types^10–15^. Similar to EGFR, insulin-like growth factor type 1 receptor (IGF-1R) and platelet-derived growth factor receptor (PDGFR) also play a major role in tumor development and progression^16,17^. Therefore, it is not surprising that inhibitors of RTKs were among the first approved targeted therapeutics in radiation oncology^18,19^. Through activation by their ligands, growth factor receptors control a network of downstream signaling molecules. One central mediator is the serine kinase AKT which is linked to DNA repair, apoptosis, protein translation, and the cellular radiation response^20–22^. In lines with this, the AKT inhibitors Ipatasertib and Capivasertib increase radio- and chemosensitivity in in vivo studies and are currently in clinical trials for targeted cancer therapy^23–25^. Further, the MAPK pathway downstream of growth factor receptors also contains several promising targets for molecular inhibitors. Trametinib targeting MEK1/2 has been approved for metastatic melanoma and is under evaluation for solid tumors harboring BRAFV600 mutations^26^.

Some of the known key driver mutations in cancer cells which are targeted by molecular inhibitors have been shown to be maintained throughout the disease course and affect tumor characteristics, radiosensitivity, and the metastatic potential^4^. However, despite several early promising results, some clinical trials found no significant benefit in adding targeted therapy to the standard-of-care cancer treatment or even observed increased normal tissue side effects^27,28^. One potential reason for these negative results is that often pre-therapeutic molecular analyses are used to select molecular therapy for treatment without taking into consideration that irradiation can alter the expression and activity of molecular targets and thereby affect the efficacy of pharmacological inhibitors^22,29^. One research focus of our group is to examine if radiation-induced target expression can be exploited to increase the efficacy of targeted drugs^22,29,30^.

The modulation of target expression can occur at both the genetic and epigenetic levels. A recent study showed that radiotherapy can increase the histone H3 methylation and lead to a stable upregulation of stem cell markers in prostate cancer cells^31^. Histone deacetylase (HDAC) inhibitors such as Pabinostat and Vorinostat affecting histone acetylation and methylation reduce tumor radioresistance and are under clinical evaluation as anti-cancer drugs^32,33^.

Building on our work with radiation-inducible molecular targets^22,29,30^, here, we show that radiotherapy impacts gene expression and protein phosphorylation of molecular targets in a fractionation- and time-dependent manner and that some of these radiation-induced changes persist for several months. Further, a 10 Gy single dose of radiation leads to an upregulation of molecular targets and increases the sensitivity of prostate cancer cells to clinically used molecular inhibitors providing a potential novel approach to using radiation plus drug treatment.

## Material and methods

### Cell culture

PC3 cells were obtained from the NCI tumor bank and used up to a passage number of 15. Asynchronously and exponentially growing cells were cultured at 37 °C and 5% CO_2_ in RPMI 1640 containing GlutaMAX (Invitrogen) supplemented with 10% fetal bovine serum (FBS, Invitrogen). Cells were regularly tested for mycoplasma contamination.

### Radiation exposure and long-term cultures

**Figure 1:**
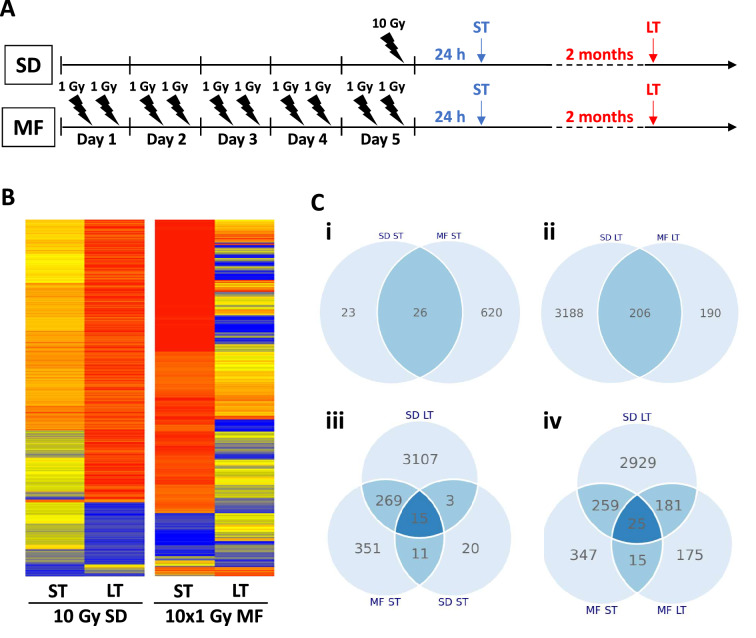
Gene expression after irradiation is fractionation- and time-dependent. (**A**) Treatment schedule for single dose (SD) and multifractionated (MF) irradiation. MF irradiation was performed with 2 fractions of 1 Gy per day to a total dose of 10 Gy. (**B**). Heat map of gene expression which was analyzed at 24 h (short term, ST) or 2 months (long term, LT) after the final irradiation dose. Relative gene expression was normalized to the unirradiated control. (**C**) Venn diagrams showing the number of overlapping or distinct significantly altered genes (fold change ≤ 0.66 or fold change ≥ 1.5, P < 0.05) under the different treatment conditions compared to the unirradiated controls.

Irradiation was performed at room temperature using single doses or multiple fractions of 320 kV X-rays with a dose-rate of 2.3 Gy/min (Precision X-Ray Inc.). Multifractionated radiation was carried out as described before with two times 1 Gy per day (with a 6 h time interval between both radiations)^34^. At 24 h after the final radiation dose or 6 d after the first radiation dose (Supplementary Figure S1), total RNA was extracted for short-term (ST) gene analysis (Fig. 1A). For long-term PC3 cultures, irradiated and unirradiated cells were passaged twice a week and cultured for at least 8 weeks after irradiation before the cells were used for long-term (LT) gene analysis or inhibitor experiments (Fig. 1A, Supplementary Figure S1).

### Colony formation assay

Colony formation assays were performed as previously described^35^. Briefly, cells were trypsinized, counted and seeded in six-well plates. Treatment with inhibitors (Table 1) was started at 24 h after plating. DMSO treated cells were used as control. The inhibitor was removed after 24 h incubation. Cells were cultured for a total of 12 days after plating. After fixation and staining with 0.4% crystal violet, cell clusters with > 50 cells were counted with a stereomicroscope (AmScope). Surviving fractions were calculated as follows: (colony number treated x cells plated untreated/ colony number untreated x cells plated treated).

**Table 1:** Overview of the inhibitors used for the experiments in Figs. 6 and 7, their main molecular targets, the disease site for which clinical trials have been performed and the clinical trial phase. The last 2 columns show the pathway activation status at 2 months after a single-dose (SD) irradiation of 10 Gy and the efficacy of the inhibitor in 10 Gy SD long-term cultures.

*Inhibitor*	Target(s)	Tumor type	Clinical trial phase	Pathway activation	Drug efficacy
Capivasertib (AZD5363)	AKT	Breast, prostate	Phase 3	+	+
Ipatasertib (GDC-0068)	AKT	Breast, prostate	Phase 3	+	+
BMS-754807	IGF-1R/InsR	Breast, metastatic solid tumors	Phase 2	+	+
Cabozantinib (XL184)	VEGFR2	Liver, thyroid, prostate, lung	Phase 4	+	+
Foretinib (GSK1363089)	MET/VEGFR2	Breast, lung, renal, HNSCC	Phase 2	+	+
Trametinib (GSK1120212)	MEK1/2	Melanoma, solid tumors	Phase 4	+	+
Ralimetinib (LY2228820)	p38 MAPK	Breast, Glioblastoma	Phase 2	+	+
Sorafenib (BAY 43-9006)	multikinase	Liver, renal	Phase 4	+	+
Dasatinib (BMS-354825)	Abl, Src, c-Kit	Leukemia, Lymphoma, prostate, lung	Phase 4	+	+
Lapatinib (GSK572016)	EGFR, ErbB2	Breast, gastrointestinal, HNSCC	Phase 4	+	unchanged
Dinaciclib (PS-095760)	CDK	Leukemia, melanoma, lung	Phase 3	+	+
Panobinostat (LBH589)	HDAC	Leukemia, multiple myeloma, lymphoma	Phase 3	−	−
Vorinostat (SAHA)	HDAC	Lymphoma, multiple myeloma, lung	Phase 3	−	−

### Whole-genome gene expression analysis

Total RNA was extracted from three replicates using a QIA shredder spin column (Catalog no. 79654, Qiagen) as published previously^34^. The RNeasy mini kit (Qiagen) was used to purify the extracted RNA. Microarray analysis was done using CodeLink Whole Genome Bioarrays representing 55,000 probes. Scanned images from arrays (gridding and feature intensity) were processed with the CodeLink Expression Analysis software (GE Healthcare), and the data generated for each feature on the array were analyzed with GeneSpring software (Agilent Technologies). Raw intensity data for each gene on every array were normalized to the median intensity of the raw values from that array.

### Pathway analysis with IPA

Activation of molecular pathways was analyzed using Ingenuity Pathway Analysis (IPA) software (Qiagen) as described before^30^. Differentially expressed genes and corresponding p values were uploaded to the IPA platform. Each gene identifier was then mapped with its corresponding gene object in the Ingenuity Pathway Knowledge Base and an activation z-score was calculated which increased or decreased depending on the known activating or inhibiting function of pathway molecules.

### Phospho-proteomic array

For analysis of the phospho-proteome, cells were plated and irradiated with 10 Gy SD, or with 10 fractions of 1 Gy dose per fraction with two fractions per day (Fig. 1A). At 30 min (ST) and at 2 months (LT) after irradiation, the cells were lysed from plates in T-Per (ThermoFisher Scientific) mixed 1:1 with 2X SDS Tris–Glycine buffer (Invitrogen, Carlsbad, CA) + 2-mercaptoethanol (final concentration = 2.5%). Reverse phase protein microarrays were performed as previously published^22^. In brief, samples were diluted and printed in duplicates onto nitrocellulose slides. HeLa cell lysates (with or without pervanadate) were used as positive and negative controls (Supplementary Figure S2). Microarrays were stained with specific and validated antibodies and analyzed with a biotin-linked signal amplification system (DAKO). The total protein amount of the sample was determined with the SYPRO Ruby stain (ThermoFisher Scientific).

### Real-time PCR

Real-time PCR was performed as previously published^29^. In brief, one microgram of total RNA was reverse transcribed using an RT2 First Strand synthesis kit (Qiagen, 330401). qPCR assays were performed using RT2 SYBR Green ROX qPCR Mastermix (Qiagen, 330520) and RT^2^ qPCR Primer Assays (Qiagen; product no. 330001) for *FGFBP1, TGFBI, PIK3CD, FGF1, PGF*, and *IGFBP1. GAPDH*, *18S*, and *Rplp0* were used as normalizing genes. Real-time PCR reactions were performed in the Applied Biosystems' thermal cycler (Quant Studio 3). PCR steps included the holding stage at 95 °C for 15 min, followed by 40 cycles of alternate denaturation at 95 °C for 15 s, annealing/extension at 60 °C for 1 min. A melt curve analysis was performed to ensure the specificity of the corresponding RT-PCR reactions. Fold change = 2-ddCt, where ddCt = dCt (test)—dCt (control); dCt = Ct (gene) – Ct (mean of *GAPDH, 18S*, and *Rplp0*); and Ct is the threshold cycle number.

### Immunofluorescence staining

Immunofluorescence was performed as recently described^29^. Cells were fixed with 3% formaldehyde/PBS for 30 min, permeabilized with 0.5% Triton X-100/PBS for 10 min and blocked with 3% BSA/PBS for 1 h. Staining of cleaved caspase 3 (Cell Signaling, #9664) was carried out overnight at 4 °C and with anti-rabbit secondary antibody for 2 h at room temperature. After several washes with PBS, samples were covered with Vectashield/DAPI mounting medium (Vector Labs). Images were acquired using an AxioImager.Z1/ApoTome microscope (Zeiss).

### Data analysis

Data were analyzed with Microsoft Excel 2019^35^. Fold change was calculated by normalizing the measured values to the corresponding control. Genes were considered upregulated if the fold change was greater than 1.5 and downregulated if the fold change was below 0.66. The unpaired, two-sided Student’s t-test was used to test for statistical significance. The irradiated samples were compared to the corresponding unirradiated controls. Results were considered statistically significant if the P value was less than 0.05.

## Results

### Fractionation impacts gene expression in a time-dependent manner

To analyze the short- and long-term effects of fractionation on the gene expression of molecular targets, we irradiated PC3 prostate cancer cells with either a single dose of 10 Gy (10 Gy SD) or a multifractionated regimen of ten 1 Gy fractions (10 × 1 Gy MF) and performed whole genome microarrays at 24 h (short-term, ST) and 2 months (long-term, LT) after the final radiation dose (Fig. 1A). Interestingly, while immediately after irradiation (ST), 10 × 1 Gy MF irradiation had a stronger impact on gene expression, we found that 10 Gy SD irradiation resulted in more long-term (LT) expression changes (Fig. 1B). From the 669 genes that were significantly (P < 0.05) upregulated (fold change > 1.5) or downregulated (fold change < 0.66) at 24 h (ST) after radiotherapy compared to the unirradiated controls, only 26 were affected by both 10 Gy SD and 10 × 1 Gy MF irradiation (Fig. 1Ci). At 2 months (LT), the expression of 206 genes was changed by both regimens, 3188 genes only by 10 Gy SD and 190 genes by 10 × 1 Gy MF irradiation (Fig. 1Cii). Further, 10 Gy SD had an impact on 18 genes at both time points (24 h-ST, 2 months-LT)(Fig. 1Ciii) and 10 × 1 Gy MF on 40 genes (Fig. 1Civ). Results showed an overlap of 284 genes which changed shortly after MF irradiation and were also differentially expressed in the long-term SD cells. Since multifractionated irradiation is delivered over 5 days in contrast to single-dose irradiation which is completed within minutes, we additionally examined the radiation-induced expression of selected genes at 6 days after start of irradiation (Supplementary Figure S1). The majority of genes showed similar expression but some genes for example *IGFBP1* were strongly upregulated after 6 days but not after 24 h (Supplementary Figure S1).

### SD results in a long-term upregulation of molecular targets

**Figure 2:**
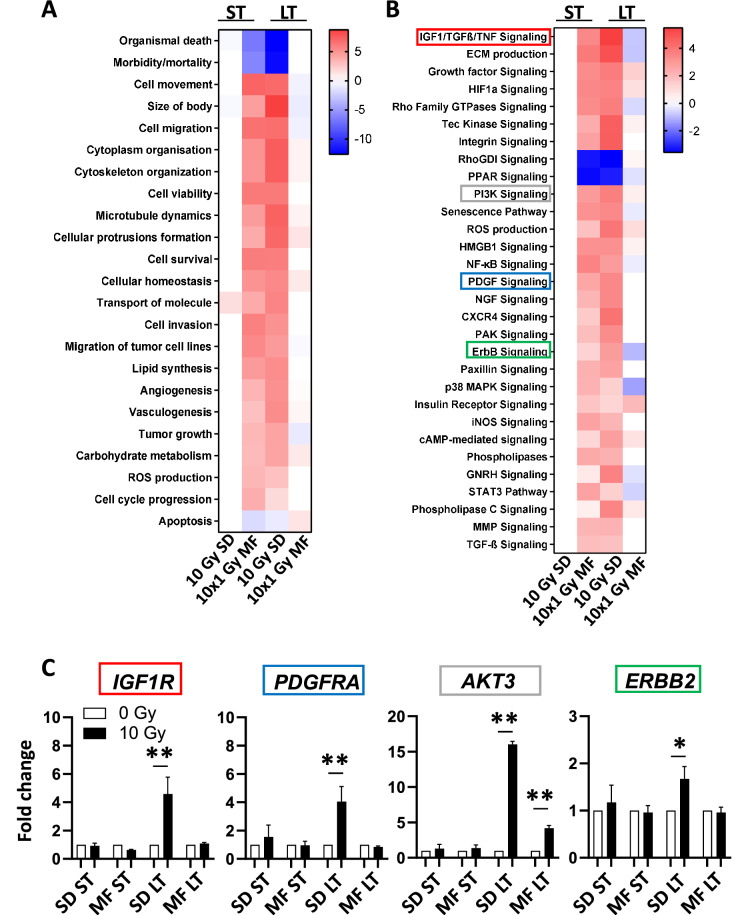
Single-dose irradiation increases the expression of molecular targets. Upregulation (red) or downregulation (blue) of (**A**) cellular functions and (**B**) molecular pathways in PC3 cells at 24 h (short-term, ST) or 2 months (long-term, LT) after a single dose of 10 Gy (10 Gy SD) or a multifractionated regimen with 10 fractions of 1 Gy (10 × 1 Gy MF) determined with the Ingenuity Pathway Analysis (IPA) software (Qiagen). (**C**) Normalized gene expression of molecular targets in PC3 cells under indicated conditions. Results show mean ± STDEV (n = 3, *P < 0.05, **P < 0.01, Student’s t-test).

Next, we examined the short- and long-term effects of irradiation on expression of genes regulating important cellular functions and pro-survival molecular pathways with Ingenuity Pathway Analysis. The pathways and cell functions which were most strongly affected by irradiation are presented in Fig. 2A and B. At 24 h (ST) after 10 × 1 Gy MF irradiation and at 2 months (LT) after 10 Gy SD irradiation, genes involved in cell movement, invasion, proliferation and survival were upregulated, while death- and apoptosis-related signaling was decreased in comparison to the unirradiated controls (0 Gy SD, 0 Gy MF)(Fig. 2A). Accordingly, growth factor-related pathways including IGF1, ErbB, PDGF, PI3K, and MAPK signaling were activated under these conditions (Fig. 2B). However, the expression of the actual target or receptor of these signaling pathways was only upregulated after 10 Gy SD but not after 10 × 1 Gy MF irradiation (Fig. 2C). It is important to note that although there were significant (P < 0.05) gene expression changes (fold change < 0.66 or > 1.5) at 24 h (ST) after 10 Gy SD irradiation compared to the unirradiated control, these did not substantially affect the activity of the selected pathways and cell functions shown in Fig. 2A and B.

### Cell functions and pathways can be activated by different gene expression patterns

**Figure 3:**
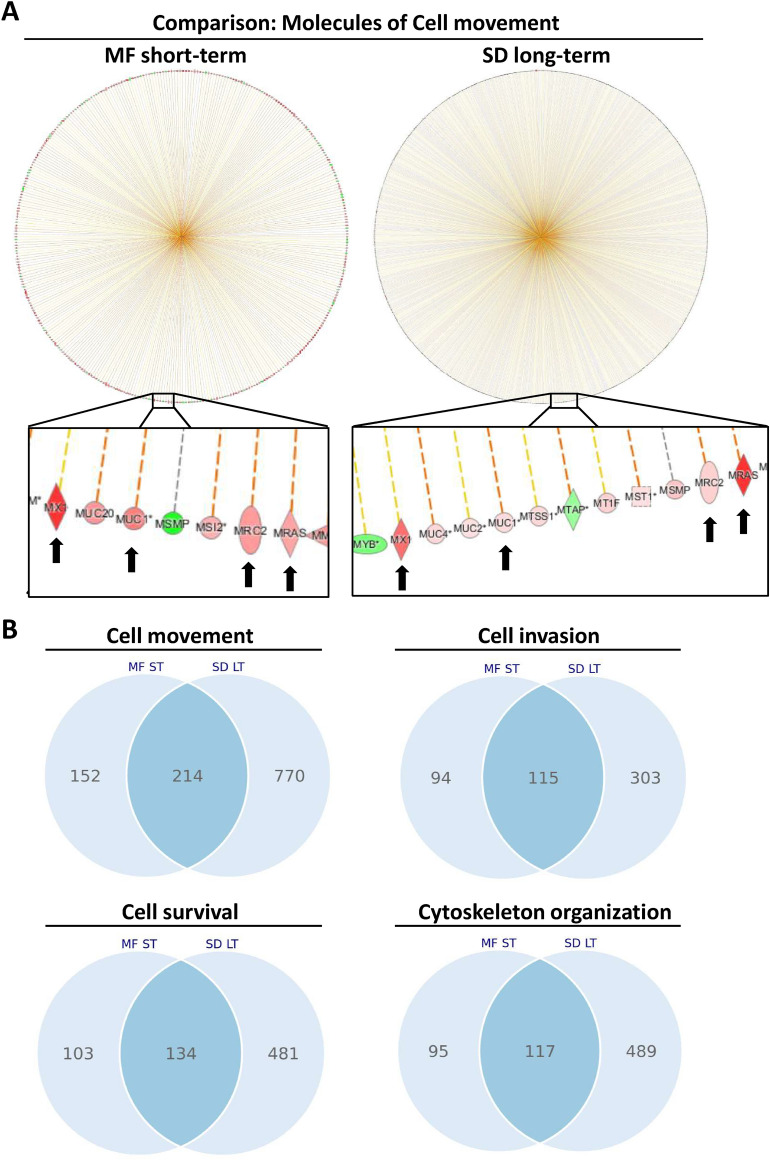
Irradiation activates pathway molecules contributing to cell movement, invasion, and survival. (**A**) Ingenuity Pathway Analysis (IPA) of genes involved in cell movement performed in PC3 cells at 24 h after a multifractionated regimen of 10 fractions of 1 Gy (MF-ST) or at 2 months after a single dose of 10 Gy (SD-LT). (**B**) Venn diagrams showing the number of overlapping or distinct significantly altered genes (fold change ≤ 0.66 or fold change ≥ 1.5, P < 0.05) involved in the indicated cell functions after a multifractionated regimen of 10 fractions of 1 Gy (MF-ST) or at 2 months after a single dose of 10 Gy (SD-LT). Gene expression has been normalized to the corresponding unirradiated control.

**Figure 4:**
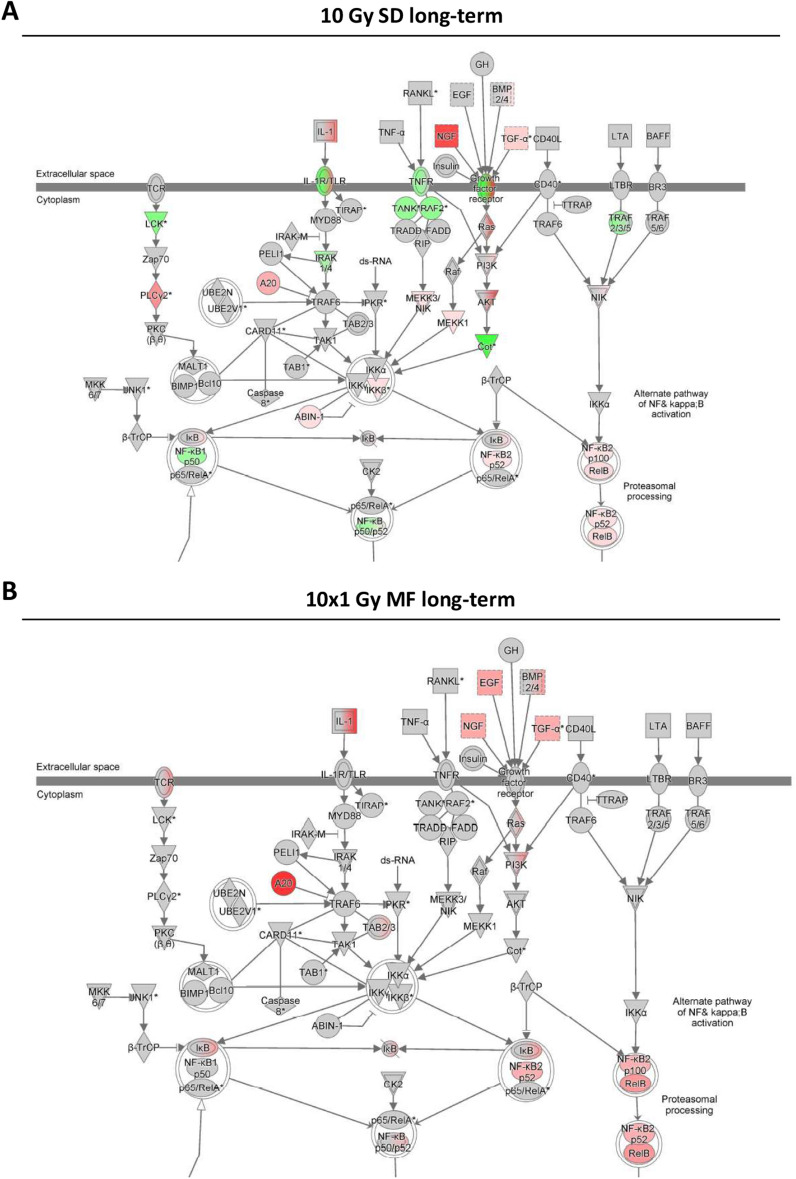
Single-dose and multifractionated irradiation activate pathways in a different manner. Gene expression in PC3 cells (**A**) at 2 months (long-term) after a single-dose (SD) irradiation of 10 Gy or (**B**) at 24 h (short-term) after a multifractionated (MF) irradiation of 10 times 1 Gy was analyzed with Ingenuity Pathway Analysis (IPA) software from Qiagen. Red color indicates upregulation, green color indicates downregulation, and white color indicates no change. Arrows and lines between molecules indicate associations and interactions.

To evaluate the similar changes in cell functions and pathways immediately after 10 × 1 Gy MF irradiation and at 2 months (LT) after 10 Gy SD further, we compared the affected genes under each condition. Although both types of irradiation led to a strong activation of cell movement, the genes causing this activation only partially overlapped (Fig. 3A, B). 214 genes were significantly (P < 0.05) altered under both conditions compared to the unirradiated controls, 152 genes were uniquely changed in PC3 cells shortly (ST) after an 10 × 1 Gy MF irradiation and 770 genes showed a differential expression in long-term (LT) 10 Gy SD cells (Fig. 3B). Similar results were obtained for other cell functions and pathways (Figs. 3B, 4).

### SD leads to a long-term increase in phosphorylation of the molecular targets AKT and MET

**Figure 5:**
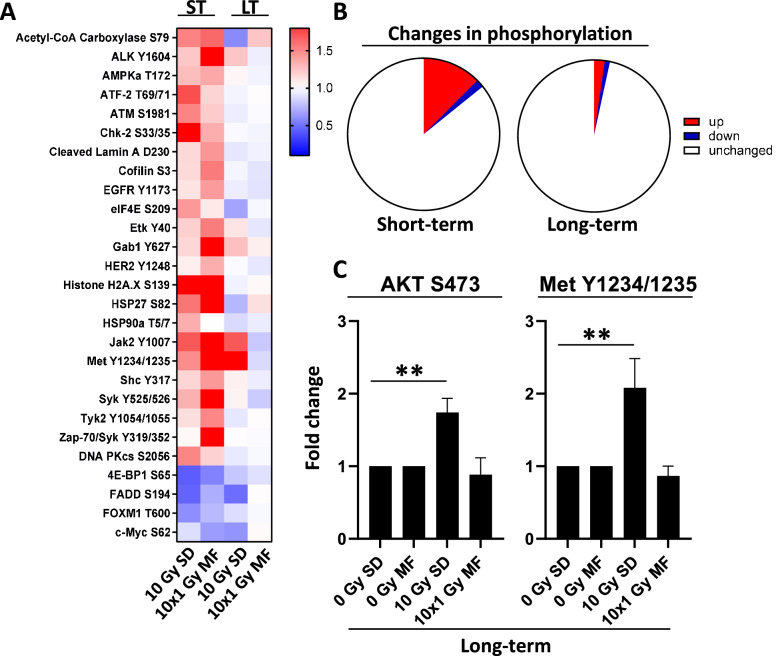
Radiotherapy induces phosphorylation immediately after the final radiation dose. (**A**) PC3 cells were irradiated either with a single dose (SD) of 10 Gy or with multifractionated (MF) irradiation of 10 times 1 Gy and incubated for 30 min (short-term, ST) or cultured for 2 months (long-term, LT). Unirradiated cells were used as control. (**B**) Significant short-term and long-term changes (fold change ≤ 0.66 or fold change ≥ 1.5, P < 0.05) in protein phosphorylation after irradiation of PC3 cells.

Since molecular targets are often regulated by protein modifications such as phosphorylation, we next examined the effects of 10 Gy SD and 10 × 1 Gy MF irradiation on the phospho-proteome (Fig. 5A). Interestingly, most of the changes in phosphorylation occurred during the first 24 h (ST) after irradiation with a peak at the 30 min time point (Fig. 5A, B, Supplementary Figure S4). Nevertheless, our analyses showed that the phosphorylation of AKT and MET was still enhanced at 2 months (LT) after 10 Gy SD indicating that irradiation can not only stably alter the expression but also the activity of molecular targets in a fractionation-dependent manner (Fig. 5C).

### Prostate cancer cells surviving SD irradiation are more sensitive to molecular targeted drugs

**Figure 6:**
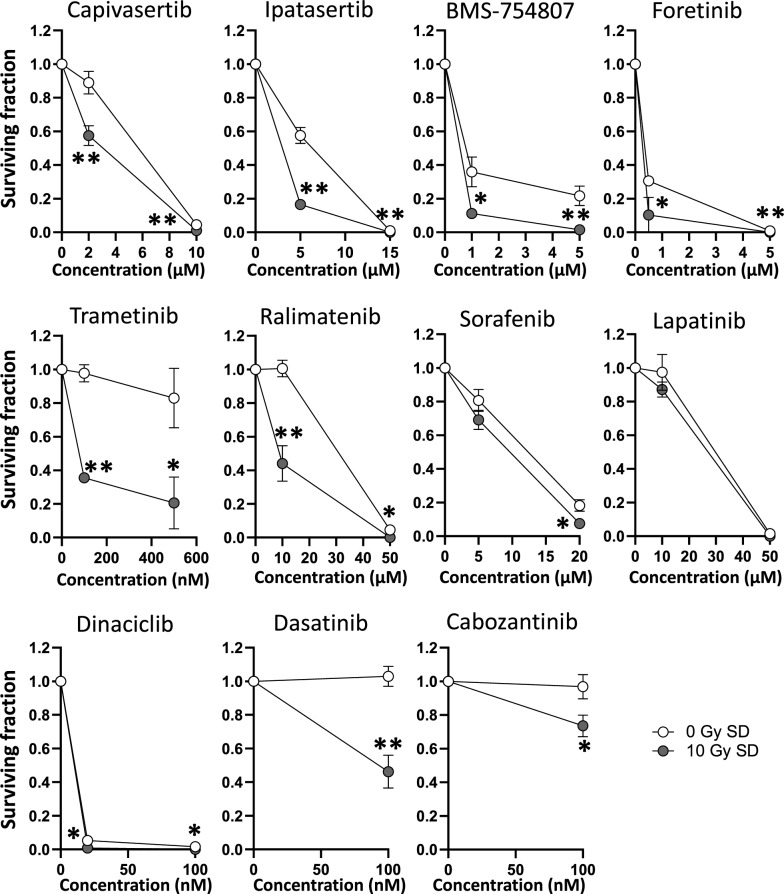
Long-term SD tumor cells are more sensitive to molecular inhibitors when the target has been activated by irradiation. PC3 cells were irradiated with a single dose (SD) of 10 Gy and cultured for 2 months. Unirradiated cells were used as control. Colony formation assays were used to determine sensitivity to molecular targeted drugs. At 24 h after plating, cells were incubated with inhibitors at indicated concentrations or DMSO (0.1%) for 24 h. Results show mean ± STDEV (n = 3, *P < 0.05, **P < 0.01, Student’s t-test).

To evaluate whether the observed overexpression and increased activation of molecular targets affects the efficacy of molecular inhibitors, we examined the sensitivity of long-term (LT) PC3 cultures to a panel of clinically used targeting drugs (Table 1). As shown in Fig. 6, PC3 cells at 2 months (LT) after 10 Gy SD irradiation showed significantly (P < 0.05) decreased survival after treatment with the AKT inhibitors Capivasertib and Ipatasertib compared to the unirradiated controls (Fig. 6). Similar results were obtained when we targeted IGF-1R, MET, VEGFR2 or MEK signaling, while 10 Gy SD irradiation had no effect on the efficacy of Lapatinib (Fig. 6). It is important to note that treatment with inhibitors induced only minimal apoptosis indicating that there might be another form of cell death as underlying mechanism for the differential survival rates (Supplementary Figure S3).

### SD irradiation reduces HDAC expression and increases the resistance of cancer cells to HDAC inhibitors

**Figure 7:**
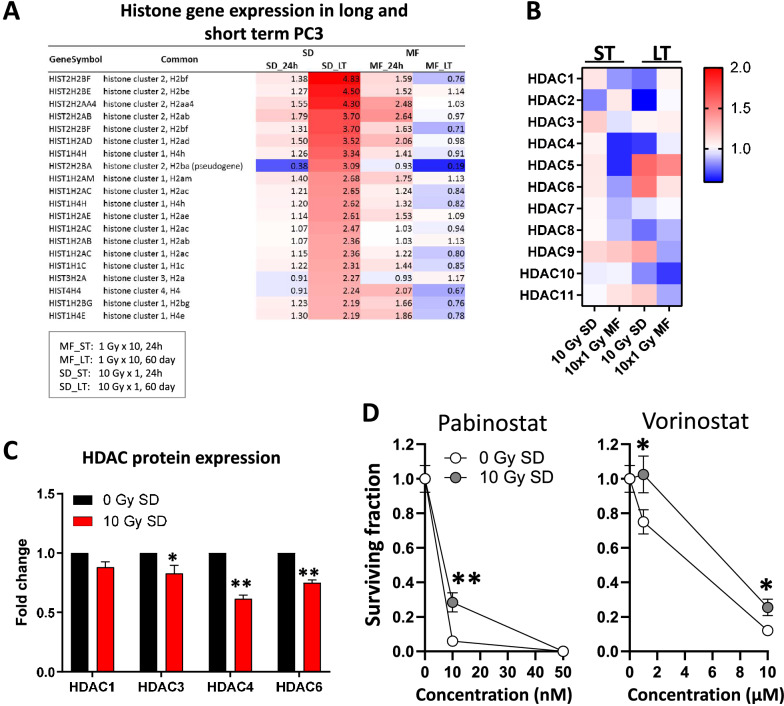
Long term SD tumor cells are more resistant to HDAC inhibition. Gene expression of (**A**) histones and (**B**) HDACs in PC3 cells at 24 h (short-term) or at 2 months (long-term) after a single-dose (SD) irradiation of 10 Gy or after a multifractionated (MF) irradiation of 10 times 1 Gy. Unirradiated cells have been used as control. (**C**) HDAC protein expression determined by reverse phase protein microarray and (**D**) clonogenic survival in long-term 10 Gy SD cells. Results show mean ± STDEV (n = 3, *P < 0.05, **P < 0.01, Student’s t-test).

As epigenetic modifications can impact gene expression, we next examined the expression of histones and HDACs after 10 Gy SD and 10 × 1 Gy MF irradiation. While at 2 months (LT) after 10 Gy SD irradiation several histone clusters were upregulated (Fig. 7A), HDAC levels were decreased (Fig. 7B, C). In parallel, long-term (LT) 10 Gy SD cells were more resistant to HDAC inhibition with Pabinostat or Vorinostat than the unirradiated controls (0 Gy SD) (Fig. 7D).

## Discussion

Recurrent disease in prostate cancer patients after curatively-intended treatment can be clinically challenging^36,37^. While after total prostatectomy conventionally fractionated radiotherapy has been shown to be effective and safe^38^, re-irradiation after prior radiotherapy carries the risk for high toxicity rates^39^. Recently, the use of stereotactic ablative irradiation with one or a few high doses has increased for both recurrent prostate cancer after prostatectomy or primary radiotherapy, as well as for metastatic disease^37,40^. Still, there is clinical need for additional therapeutic strategies to improve patient outcome after disease relapse. During the last two decades, targeted therapy has evolved as promising approach for multiple cancer types for either monotherapy or in combination with irradiation or chemotherapy resulting in improved tumor response and patient survival^41^. However, some tumors have an intrinsic resistance to targeted therapy or develop resistance during treatment^41^. Since radiotherapy can modulate gene expression and protein phosphorylation, this effect can potentially be exploited to increase or restore the efficacy of targeted therapy^22,42^. Using the post-radiation adaptation of tumors to enhance efficacy of radiation therapy is different and complementary to using radiation and drugs simultaneously^30,42,43^. We demonstrate here that irradiation leads to long-term expression changes of multiple molecular targets and their associated pathways in surviving prostate cancer cells and that these adaptive changes are impacted by the time interval and fractionation regimen. It is important to note that both SD and MF irradiation regimens differ in their biologically equivalent doses (BED), although they have the same total radiation dose^44^. The BED is based on the linear quadratic (LQ)-model and functions as a parameter for the biological effect of irradiation by taking into account the dose-per-fraction, total dose and treatment time^45^. It has been shown that the BED can affect gene expression and therefore may contribute to the differential results between SD and MF irradiation which we observed^45^.

Among others, IGF signaling was strongly activated at 2 months after 10 Gy SD but not after 10 × 1 Gy MF. Interestingly, high IGF-1R expression has been associated with high prostate cancer recurrence after primary radiotherapy indicating a potential role for IGF-1R for the adaptive tumor response^46^. Further, inhibition of IGF-1R sensitizes cancer cells to chemotherapy and irradiation identifying it as a promising target for molecular therapy^47,48^. Besides the higher IGF-1R expression, long-term 10 Gy SD cultures were also more sensitive to treatment with the IGF-1R inhibitor BMS-754807 which is in line with observations from Litzenburger and colleagues showing a correlation between IGF-1R expression and BMS-754807 efficacy in triple-negative breast cancer cell lines^49^. In contrast to targets such as IGF-1R and AKT3, SD irradiation reduced the expression of HDACs and in parallel increased the resistance to the HDAC inhibitors Pabinostat and Vorinostat. By modulating histone acetylation and methylation, targeting HDACs has been shown to radiosensitize cancer cells even when the inhibitors are applied up to 24 h after irradiation^32,50,51^. Interestingly, fractionated irradiation of 10 × 2 Gy doses results in elevated HDAC activity in breast cancer cells at 21 days after the final dose which correlated with enhanced cellular radioresistance indicating that HDACs are affected by irradiation depending on the fractionation regimen^52^.

Similar to gene expression, protein phosphorylation of the target also strongly affects the efficacy of targeted therapy and can be exploited to sensitize resistant cancer cells^22^. While we saw more pronounced modulation of gene expression at later time points, the radiation-induced alterations in protein phosphorylation occurred mainly within the first 24 h after irradiation and were more often transient than permanent. Targets showing increased long-term phosphorylation and activation included the kinases AKT and MET. Elevated phosphorylation of AKT promotes survival and radiation resistance and is a negative prognostic marker for poor clinical outcome in prostate cancer patients^20–22,53,54^. Combined treatment with the AKT inhibitor Ipatasertib and abiraterone significantly increased progression-free survival of metastatic castration-resistant prostate cancer patients compared to abiraterone alone especially in patients with PTEN-loss tumors and activated PI3K/AKT signaling^55^. Similar results were found in a randomized phase II trial examining AKT inhibition with Capivasertib in combination with Paclitaxel for women with metastatic breast cancer indicating that stimulating AKT activity before targeting it may increase the drug efficacy^56^.

Overall, our data show that radiotherapy especially large single doses can lead to stably elevated gene expression and activity of molecular targets and hereby sensitize cancer cells to the corresponding pharmacological inhibitors. Since the use of stereotactic ablative radiotherapy applying one or a few high doses has substantially increased, this may be a unique approach to improve the therapy outcome for recurrent and locally advanced prostate cancer patients.

## Supplementary Information

Supplementary Information.
