# Supplementary material for: Radiotherapy alters expression of molecular targets in prostate cancer in a fractionation- and time-dependent manner
Source: Sci Rep. 2022 Mar 3;12:3500. doi: 10.1038/s41598-022-07394-y (PMC8894377; doi:10.1038/s41598-022-07394-y)
Supplement: Supplementary file 1 — Supplementary Information. [file 41598_2022_7394_MOESM1_ESM.pdf]

Supplementary Figure S1

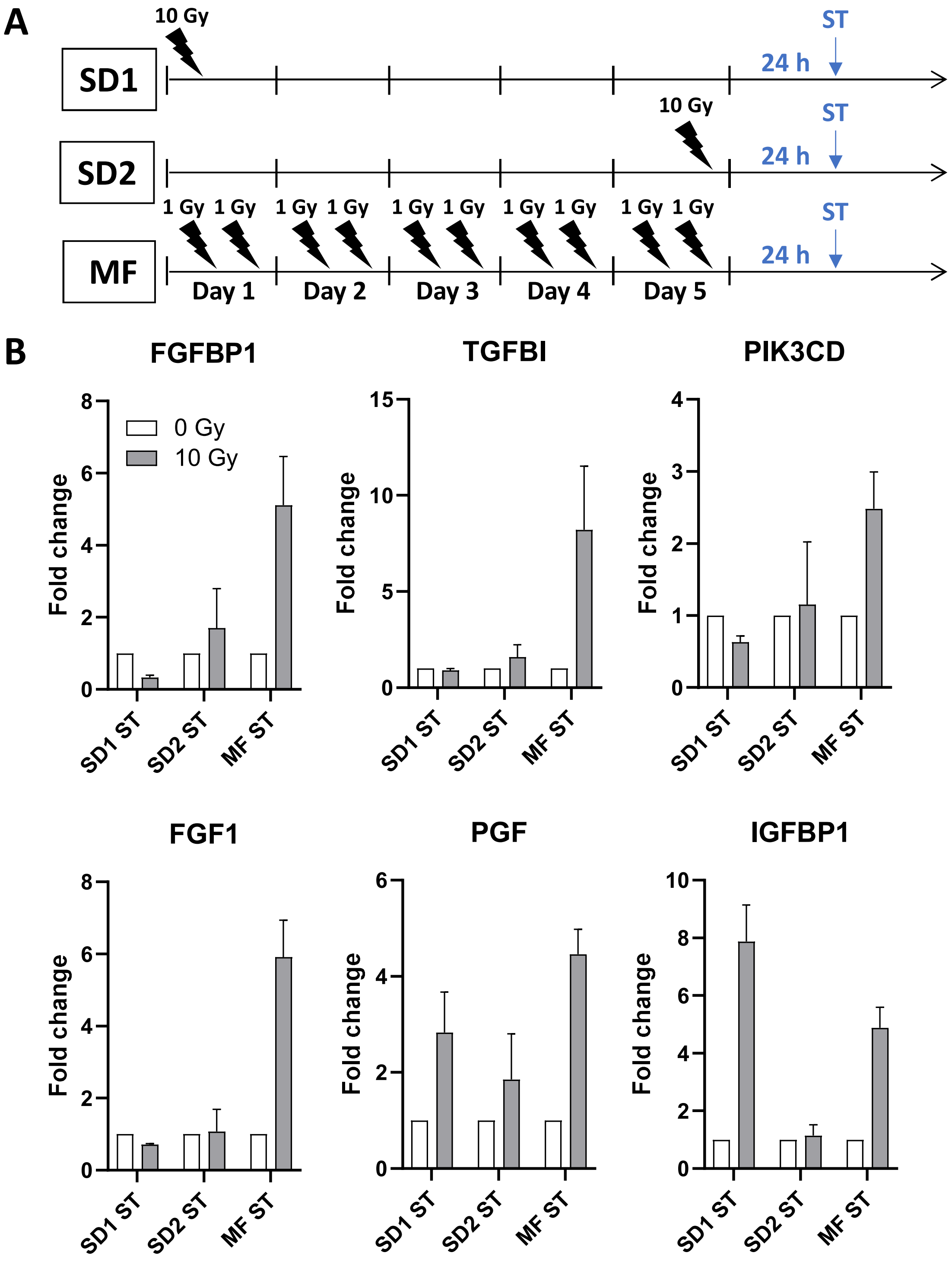

(A) PC3 cells were plated and irradiated with 10 Gy SD, or with 10 fractions of 1 Gy dose per fraction with two fractions per day. At 6 days after first radiation dose (SD1, MF) and at 24 h after final irradiation dose (SD2, MF), gene expression of indicated genes was analyzed.

Supplementary Figure S2

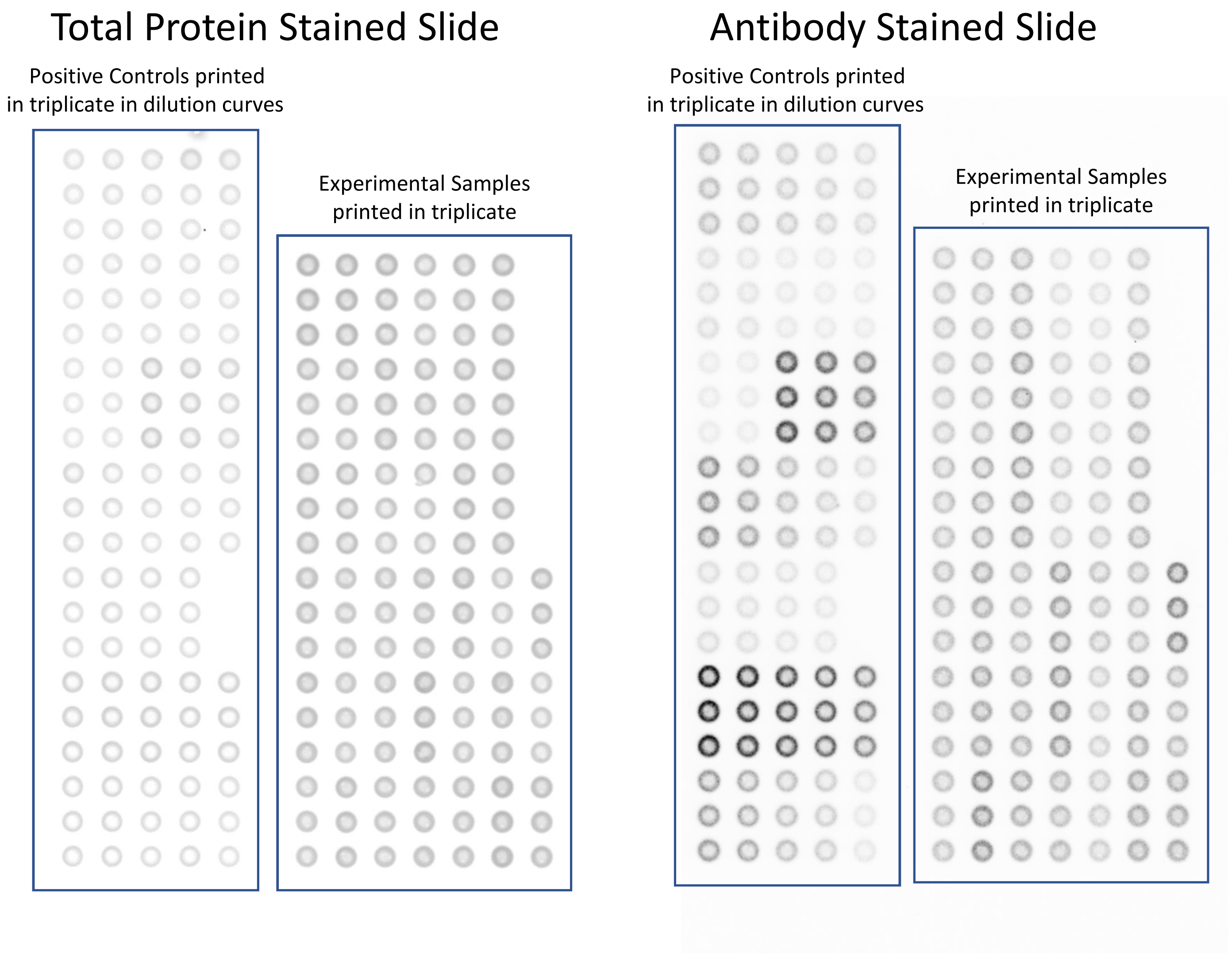

PC3 cells were plated and irradiated with 10 Gy SD, or with 10 fractions of 1 Gy dose per fraction with two fractions per day (Figure 1A). At 30 min and at 2 months after irradiation, cells were lysed with 2.5% 2-mercaptoethanol in T-PER including protease and phosphatase inhibitors. Samples were diluted and printed in duplicates onto nitrocellulose slides. HeLa cell lysates (with or without pervanadate) were used as positive and negative controls. Microarrays were stained with specific and validated antibodies and analyzed with a biotin-linked signal amplification system.

Supplementary Figure S3

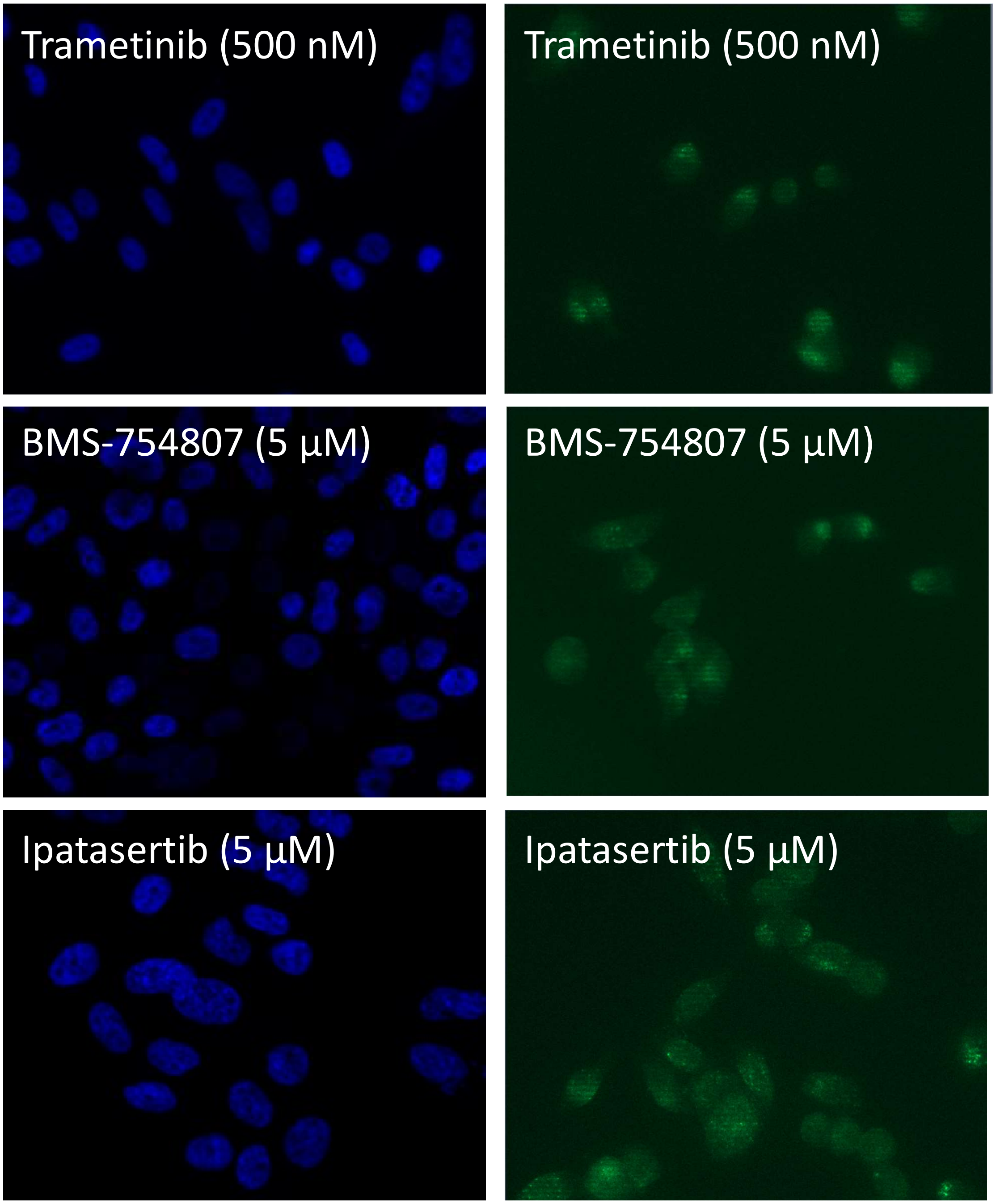

PC3 cells were irradiated with a single dose (SD) of 10 Gy and cultured for 2 months. At 24 h after plating, cells were incubated with inhibitors at indicated concentrations. Samples were fixed after 48 h for DAPI staining or 8 h for Cleaved Caspase-3 (Asp175) (5A1) Rabbit mAb (Cell Signaling) staining. Images were acquired using an AxioImager.Z1/ApoTome microscope (Zeiss).

Supplementary Figure S4

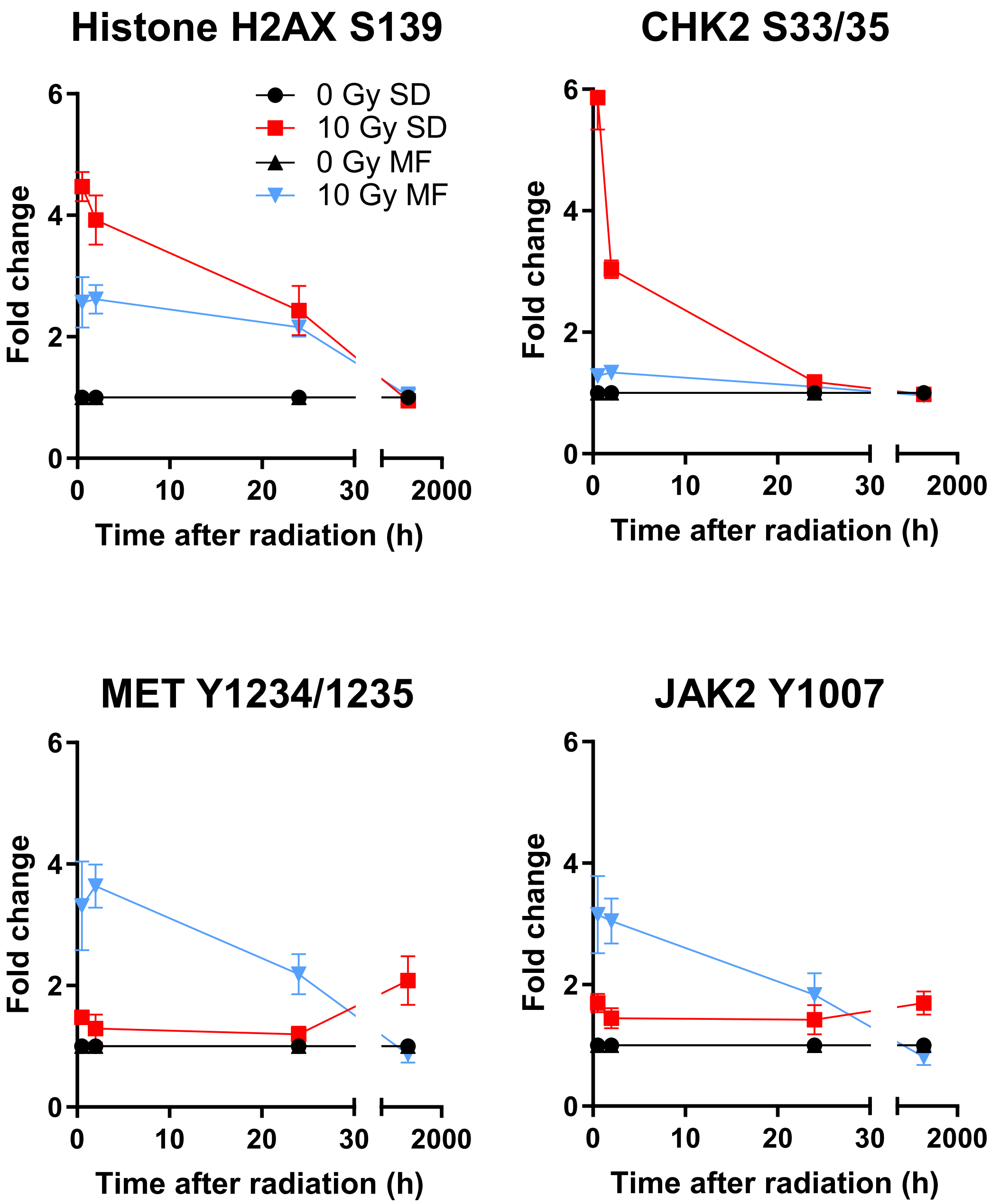

PC3 cells were irradiated either with a single dose (SD) of 10 Gy or with multifractionated (MF) irradiation of 10 times 1 Gy and incubated for 30 min, 2 h, 24 h, or cultured for 2 months. Unirradiated cells were used as control. At indicated time points, cells were lysed with 2.5% 2-mercaptoethanol in T-PER. Protein phosphorylation was evaluated with reverse phase protein microarrays.
